# Status epilepticus associated with platinum chemotherapy in a patient with cervical cancer: a case report

**DOI:** 10.1186/s12885-015-1755-2

**Published:** 2015-10-17

**Authors:** Laura L. Holman, Yulan Ren, Shannon N. Westin

**Affiliations:** 1 Department of Gynecologic Oncology and Reproductive Medicine, The University of Texas MD Anderson Cancer Center, Unit 1362, 1155 Herman Pressler, CPB6.3279, Houston, TX 77030-3721 USA; 2 Department of Gynecologic Oncology, Fudan University Shanghai Cancer Center, 159 Tianzhou Road, Xuhui Area, Shanghai, 200030 China

**Keywords:** Platinum chemotherapy, Seizure, Neurotoxicity, Cervical cancer

## Abstract

**Background:**

While peripheral neuropathy is a common side effect of platinum-based chemotherapy, central nervous system (CNS) toxicity, such as encephalopathy or seizures, appears to be rare. This manuscript describes the only reported case of nonconvulsive status epilepticus associated with cisplatin in a cervical cancer patient who does not have other underlying medical conditions.

**Case presentation:**

The patient is a 54-year-old Chinese female with recurrent stage IIIB moderately differentiated squamous cell carcinoma of the cervix who was being treated with cisplatin and topotecan. During the sixth cycle of this regimen, the patient presented with mental status changes. While imaging and laboratory values were within normal limits, the patient’s EEG revealed nonconvulsive status epilepticus. After appropriate intervention, she made a complete recovery with no further seizures. The patient currently remains on antiepileptic therapy, but is no longer receiving cisplatin.

**Conclusion:**

Patients who present with new onset seizures should primarily be evaluated for underlying medical conditions. Among patients who are suspected to have CNS side effects associated with platinum use, we recommend avoidance of platinum agents in future chemotherapeutic regimens. Although rare, providers should be aware of the potential for CNS toxicity associated with this drug class.

## Background

Platinum analogs are chemotherapeutic agents used to treat a wide variety of malignancies. Members of this drug class commonly prescribed for gynecologic malignancies include cisplatin and carboplatin. These medications work by covalently binding to DNA to produce intrastrand and interstrand crosslinks. The formation of these DNA adducts leads to inhibition of transcription and replication, as well as cell-cycle arrest [1].

The most common side effects associated with platinum agents are nephrotoxicity, myelosuppression, nausea and vomiting, and hypersensitivity reactions. Neurotoxicity is also reported, though the frequency varies widely among the drugs in this class. While up to 60 % of patients receiving cisplatin have neurotoxicity, only 4-5 % of patients who receive carboplatin experience this side effect [2].

Peripheral neuropathy is the most common neurological toxicity associated with platinum agents. Patients with platinum-induced peripheral neuropathy can experience paresthesia in a “glove and stocking” distribution, loss of vibratory sensation, and loss of proprioception that can severely affect activities of daily living and quality of life. Ototoxicity, in the form of hearing loss or tinnitus, is also a well-described neurological side effect associated with cisplatin. Encephalopathy, transient blindness, aphasia, and seizures are reported to be associated with platinum use, but appear to be exceedingly rare in the absence of brain pathology, laboratory abnormalities, or medication overdose [3–5].

In this paper, we report a case of status epilepticus associated with cisplatin in a patient with recurrent cervical carcinoma.

## Case presentation

The patient is a 54-year-old female with recurrent stage IIIB moderately differentiated squamous cell carcinoma of the cervix. Patient had no relevant prior medical or family history. Her initial treatment included one cycle of oxaliplatin and tegafur followed by intensity-modulated radiotherapy (IMRT) and vaginal brachytherapy. At completion of her radiation, the patient received five cycles of taxane and platinum-based chemotherapy with a complete response. She remained without evidence of disease for two years, when she was diagnosed with a recurrence in her retroperitoneal and thoracic lymph nodes. Oxaliplatin and paclitaxel were initiated, but discontinued after one cycle due to vague “mental status changes.” She underwent brain imaging at that time that was within normal limits. She was then treated with radiation to the retroperitoneal lymph nodes followed by immunotherapy with dendritic cell and cytokine-induced killer cell transfusions resulting in a partial response. The patient was then initiated on an oral taxane for two cycles with persistent disease. She then presented to our institution and was dispositioned to topotecan (0.75 mg/m^2^) daily on cycle days one, two, and three and cisplatin (50 mg/m^2^) on cycle day 1, administered every three weeks. The patient tolerated the first five cycles without incident and achieved an objective tumor response. However, on day two of cycle six, she developed altered mental status and was not responding appropriately to questions or commands. She was brought to the emergency room where vital signs and laboratory values, including complete blood count, complete metabolic panel, and urinalysis, were within normal limits. Neurological examination revealed no focal deficits. She underwent CT and MRI of the brain, which were also within normal limits. However, electroencephalogram (EEG) revealed the presence of nonconvulsive seizures (NCS). She was given a one-time dose of lorazepam with significant improvement in her mental status. The patient was also initiated on levetiracetam. A repeat EEG three days later noted resolution of the seizure activity. After this episode, the patient was transitioned to topotecan and bevacizumab with no further seizures to date. Please reference Table 1 for timeline of events.

**Table 1:** Timeline of events

Date	Key Event
July 2011	Diagnosis of Cervical Cancer
August 2011 – January 2012	Primary Treatment
	-oxaliplatin/tegafur
	-IMRT/brachytherapy
	-Paclitaxel/platinum
January 2013	Recurrence of disease
January 2013 – October 2013	Treatment for Recurrence
	-oxaliplatin/paclitaxel
	- tumor-directed radiation
	- dendritic cell immunotherapy
	- taxane therapy
October 2013	Progression of disease/consultation at MD Anderson Cancer Center
October 2013	Initiation of topotecan/cisplatin
October 2013 – January 2014	Cycles #1–5 of topotecan/cisplatin
February 2014	Cycle #6 of topotecan/cisplatin
	SEIZURE ACTIVITY

## Conclusions

This case describes the unusual presentation of a patient in nonconvulsive status epilepticus attributed to cisplatin use. As the patient had normal laboratory and imaging findings without convulsions, the diagnosis was somewhat more challenging. Interestingly, the patient reported a history of vague mental status changes with platinum use in the past. On further questioning, it appears that her symptoms at that time were very similar to her current presentation. However, she did not undergo EEG evaluation or further neurological work-up.

While central nervous system (CNS) disorders are not frequently associated with platinum use, cases have been reported [3–6]. Posterior reversible encephalopathy syndrome (PRES) is the most commonly described platinum-associated CNS toxicity in the literature [7]. PRES can occur in a wide variety of clinical settings, including eclampsia, sepsis, autoimmune disease, and medication-related toxicity. Symptoms of PRES typically involve headache, visual changes, seizures, and encephalopathy. PRES is diagnosed by clinical presentation as well as brain MRI, where white matter edema of the posterior cerebral hemispheres is typically seen. When PRES is associated with platinum drugs, symptoms have been reported to occur within a few hours of cisplatin administration and typically resolve in a few days [6].

Several authors have described the presence of seizures after platinum administration not attributable to PRES, including two cases of status epilepticus [7–9]. However, almost all of these patients had laboratory derangements such as hypomagnesemia or renal insufficiency, or imaging abnormalities such as brain metastases, which could lower the seizure threshold. In the absence of other medical factors that could potentiate them, seizures associated with platinum chemotherapy appear to be exceedingly rare. This manuscript describes the only reported case of nonconvulsive status epilepticus related to cisplatin use in a cervical cancer patient without other underlying medical conditions.

The etiology of platinum-induced CNS toxicity is unknown, though heavy metal toxicity or demylinization have been proposed [7]. Overdose of platinum agents can certainly also cause these disorders. However, reports have noted CNS toxicity with these drugs at a wide range of doses and in combination with various chemotherapeutic agents. As platinum agents do not typically cross the blood-brain barrier, patients should be protected from most CNS side effects at doses in the therapeutic range. It is unclear why this is not true for all patients.

In conclusion, CNS toxicity appears to be a rare but serious side effect of administration of platinum-containing drugs. Patients who present with CNS symptoms should primarily be evaluated for other underlying medical conditions. However, if platinum-based chemotherapy is suspected as the etiology, we suggest the use of alternative chemotherapeutic regimens in the future.

## Consent

Written informed consent was obtained from the patient for publication of this case report and any accompanying images. A copy of the written consent is available for review by the Editor of this journal.

## Abbreviations

CNS: Central nervous system
EEG: Electroencephalogram
IMRT: Intensity-modulated radiotherapy
NCS: Nonconvulsive seizures
PRES: Posterior reversible encephalopathy syndrome
